# Reversibly pH-responsive gold nanoparticles and their applications for photothermal cancer therapy

**DOI:** 10.1038/s41598-019-56754-8

**Published:** 2019-12-27

**Authors:** Sanghak Park, Woo Jin Lee, Sungmin Park, Doowon Choi, Sungjee Kim, Nokyoung Park

**Affiliations:** 1 Department of Chemistry, Myongji University, 116 Myongji Ro, Yongin, Gyeonggi-do 17058 South Korea; 2 Department of Chemistry, POSTECH, 77 Cheongam Ro, Nam Gu, Pohang 37673 South Korea

**Keywords:** Biotechnology, Chemistry, Materials science, Nanoscience and technology

## Abstract

Microenvironment responsive nanomaterials are attractive for therapeutic applications with regional specificity. Here we report pH responsive gold nanoparticles which are designed to aggregate in acidic condition similar to cancer environment and returned to its original disassembled states in a physiological pH. The pH responsive behavior of the particles is derived by change of electrostatic interaction among the particles where attraction and repulsion play a major role in low and high pH of the environment, respectively. Since different electrostatic interaction behavior of the particles in varied pH is induced not by irreversible chemical change but by simple protonation differences, the pH responsive process of assembly and disassembly is totally reversible. The low pH specific aggregation of gold nanoparticles resulted in red shift of plasmonic absorption peak and showed higher photothermal efficacy in acidic pH than in normal physiological pH. The low pH specific photothermal effect with long wave laser irradiation was directly applied to cancer specific photothermal therapy and resulted higher therapeutic effect for melanoma cancer cells than non-pH responsive gold nanoparticles.

## Introduction

The optical properties of gold nanoparticles (AuNPs) have been greatly attractive since their useful biological and medical applications such as CT contrast Raman imaging, and radio-sensitization^1–6^. Especially, the highly efficient photothermal effect and relative bio-safety of gold nanoparticles have driven extensive studies for noninvasive cancer therapeutic applications^7–9^. As hyperthermia agents, diverse structures of AuNPs including spheres, rod, branched shapes and cages have been developed^10–13^. For higher therapeutic efficiency of the AuNPs for cancers in deep tissue, it has been important to improve the resonant absorption for near-infrared (NIR) which has superior tissue penetration depth^14,15^. The absorption properties such as peak absorption wavelength or absorptivity of AuNPs depend on their size or three-dimensional structures^16^. In terms of the size of AuNPs, relatively large sizes (>100 nm) are required to achieve the high absorption for NIR^17,18^. The application of large particles, however, are limited since the larger the particles are less advantageous to be excreted from the body and the accumulated particles eventually have a potential to be toxic^19,20^. Another well-known method to achieve high NIR absorption is to fabricate AuNP structures with high aspect ratios such as nanorods, urchin-like or branched shapes. The fabrication process of such high aspect ratios of AuNP nanostructures often required complicated processes and also toxic chemicals such as detergents. To overcome the discrepancy between desired plasmonic properties and biological safety of those particles, aggregated or assembled systems of small AuNPs utilizing their coupled surface plasmons in closely located AuNPs have been proposed as alternatives^21^. Most of those proposed methods, however, employed polymers or lipids to induce nanoparticle aggregations resulting restricted functionalization^22,23^, limited payload for the AuNP and other cargos, and more importantly irreversible AuNP agglomeration. We have previously reported a reversible AuNP assembly system for synergistic cancer therapy which is light responsive^24^. However, this reversible system also has had a limitation in terms of cancer targeting since the AuNPs were pre-assembled on a DNA hydrogel scaffold in a tube and directly injected to the tumor cells. Here we propose newly designed AuNPs those can aggregate selectively in tumors by responding to relatively low pH which is specific property of cancer shifting the plasmonic absorption to far-red and reversibly disassemble when the particle clusters meet a normal physiological pH around 7.4^25^.

## Results and Discussion

**Figure 1:**
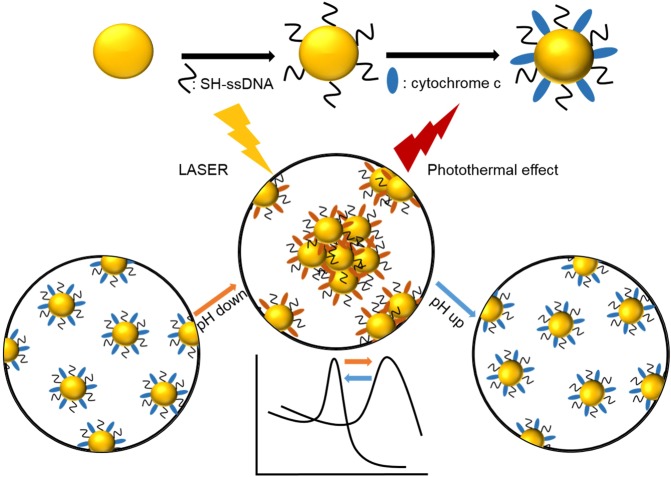
Schematic diagram of surface modification process of AuNPs with ssDNA and cytochrome c modification process (upper) and pH responsive behavior of CytC/ssDNA-AuNPs (lower).

pH-responsive AuNPs have been synthesized by introducing mixed layer of single stranded DNA and cytochrome c on the surface of AuNPs^26,27^. For the synthesis of pH-responsive AuNPs, 10 nm in diameter of spherical gold nanoparticles are used and this small size is advantageous for fast excretion from a body. The DNA has been chosen to form a consistently negative charged layer in the experimental pH range of 8.0 ~ 5.0 considering the strands’ relatively low isoelectric point (pI) of around 4.0~4.5^28^. In the contrast, the layer of cytochrome c has been employed as a zwitterionic moiety with pI of around 9.6 resulting increase of the positively charged portions of the layer with the reducing the experimental pH from 7.4 to 5.5 or reversibly resulting decrease of positive portion by raising the pH values^29^. The stepwise synthesis of CytC/ssDNA-AuNPs has been characterized by measuring plasmonic absorption peak shift, their size distribution and zeta potential in solution. Mobility change in agarose gel electrophoresis has been also used to confirm the surface modification of the nanoparticles (Fig. S1). The synthesized CytC/ssDNA-AuNPs showed stability good enough to be used for next experiments in various buffer solutions as well as in deionized water (Fig. S2). With those modified surfaces, in a normal physiological pH (~7.4), the surface charge of AuNP is negative enough to repel each other due to electrostatic repulsion and the AuNPs exist individually within a cell. The charge of cytochrome c on AuNP surface, however, gradually changed to be positive with reduced pH (<6.5) and eventually the AuNPs aggregated forming large clusters by local electrostatic attraction which is induced by the opposite charge of DNA strands and cytochrome c (Fig. 1).

**Figure 2:**
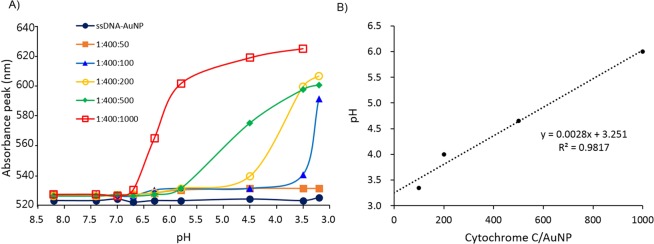
(**A**) Absorption peak wavelengths of CytC/ssDNA-AuNPs in various pHs. The numbers on the symbols represent the ratios of AuNPs vs. ssDNA vs. cytochrome c of the modification reaction. (**B**) Responsive pHs vs. reaction ratio of cytochrome c over AuNPs of each CytC/ssDNA-AuNP.

The responsive pH values, here we define it as pH values at the inflection points of the plasmonic absorption peaks vs. pH curves, of the CytC/ssDNA-AuNPs can be easily tuned by simple control of mixing ratio of cytochrome C and ssDNA during AuNP surface modification reaction. The varied mixing ratio of ssDNA and cytochrome c during the surface modification reaction gave different ratios of the number of those surface ligands on each AuNPs (see Fig. S4 and Table S1). During the modification reaction, the ratios of AuNP vs. ssDNA vs. cytochrome c were varied from 1: 400: 0 to 1: 400: 1000. As shown in Figs. 2 and S3, the aggregation pH values were raised with increasing the relative amount of cytochrome c over ssDNA. This responsive pH tuning ability has a potential to be applied for regional pH mapping in acidic tumor cells as well as efficient targeting of cancer cells. The CytC/ssDNA-AuNPs synthesized at the ratio of 1: 400: 1000 (AuNP: ssDNA: cytochrome c) were used for all of the following experiments.

**Figure 3:**
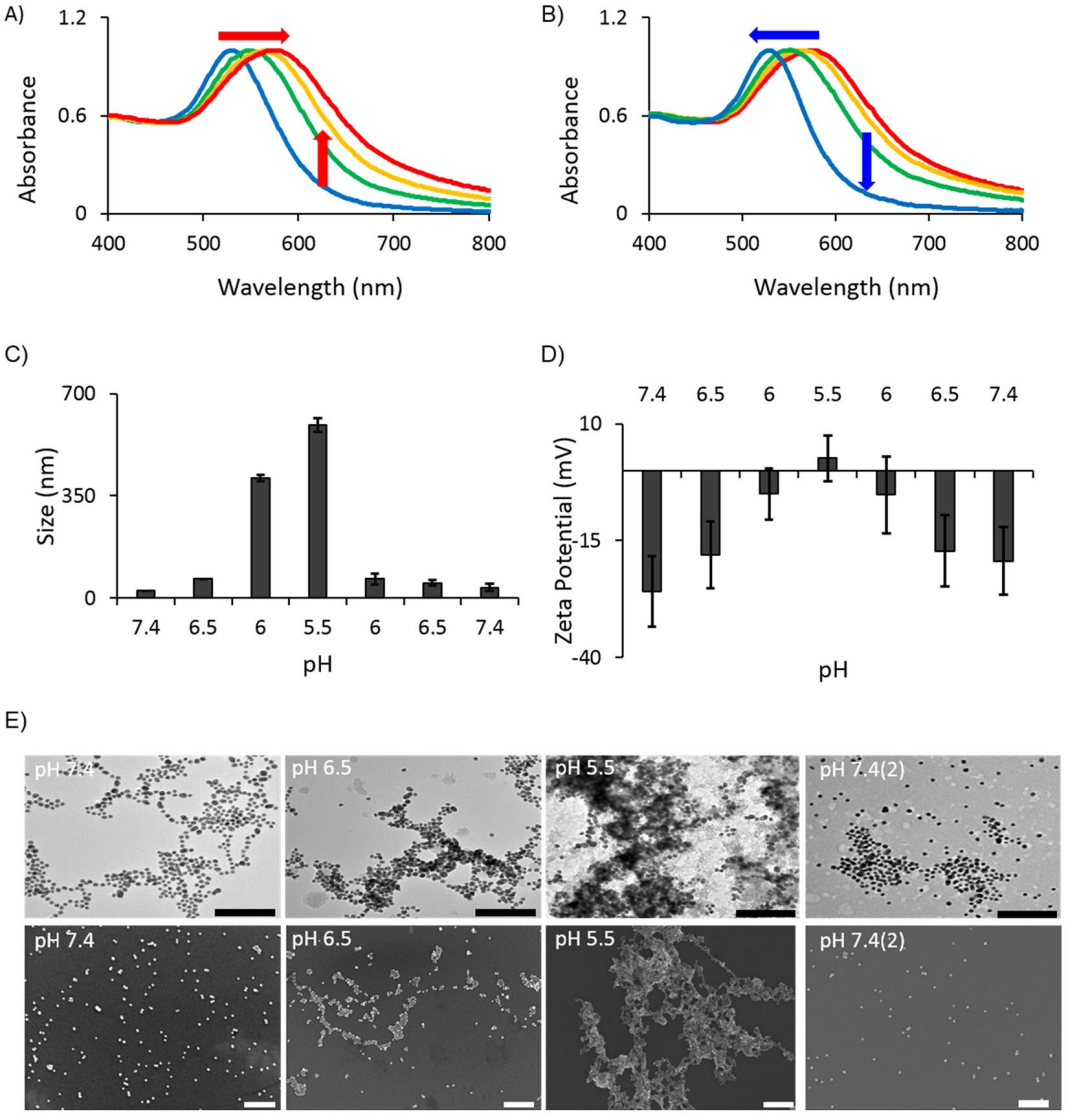
(**A**) Red shift of absorption peak accordingly with reducing pH (7.4, 6.5. 6.0 to 5.5). (**B**) Blue shift of absorption peak with returning to higher pH (5.5, 6.0, 6.5 to 7.4). (**C**) Sizes, and (**D**) Zetapotentials of CytC/ssDNA-AuNPs measured during the pH lowering and elevating process. (E) TEM (upper panel) and SEM (lower panel) images of CytC/ssDNA-AuNPs taken at a cycle of pH 7.4 → 6.5 → 5.5 → 7.4 (scale bar: 300 nm). Error bars refer to standard deviations from three replicates.

pH responsiveness of the synthesized AuNPs (CytC/ssDNA-AuNP) has been confirmed by measuring plasmonic absorption peak shift, size of clusters, and taking SEM images at various pH values. As shown in Fig. 3, Tables S1 and S2, the various physical properties of CytC/ssDNA-AuNPs have changed accordingly with the solution pH values and the changes have been also reversible. The plasmonic absorption peak has shifted to a longer wavelength implying that the particles aggregated to larger plasmonic clusters when the pH was reduced to 5.5 which is similar to the characteristic pH value of cancer cells (Fig. 3A). Remarkably, the absorption peak reversibly blue shifted to its original one accordingly with the pH restoring to 7.4 which is normal physiological condition (Fig. 3B). This means that the aggregated nanoparticles in low pH have been disassembled and back to individually existing status. Considering there is no significant pH variation-associated response of ssDNA-AuNPs, which is modified with only single stranded DNA without cytochrome c on the particle surfaces (Fig. S3), the pH responsive behavior of CytC/ssDNA-AuNPs can be regarded as a result of the surface charge variation influenced by combined electrical properties of cytochrome c and ssDNA as expected in above description and Fig. 3C. The pH responsiveness of CytC/ssDNA-AuNP has been consistently confirmed by measuring the particle size in the solution and taking SEM images (Fig. 3D,E). According to the dynamic light scattering experiment, the size of the AuNPs in a solution has been increased from 12 nm to larger than 600 nm in diameter with the reducing pH from 7.4 to 5.5. The increased size of AuNPs is attributed to the electrostatic clustering of the nanoparticles due to the decreased zeta potentials at low pH.

**Figure 4:**
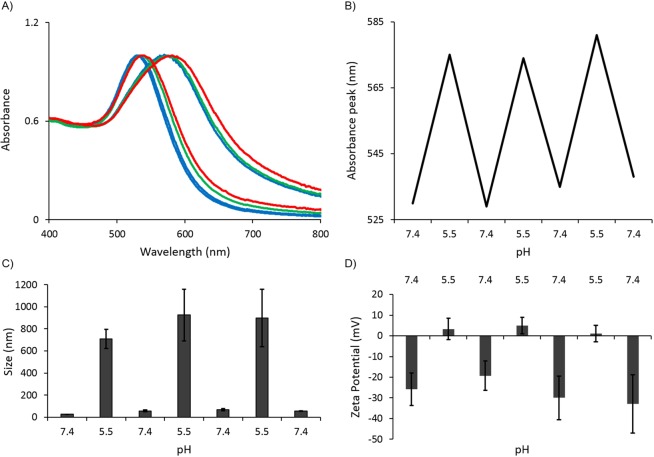
(**A**) Reversible absorption spectrum shift of CytC/ssDNA-AuNPs accordingly with pH variations. Blue lines: 1^st^ cycle of pH 7.4 to 5.5 to 7.4, Green lines: 2^nd^ cycle of pH 5.5 to 7.4, and Red lines: 3^rd^ cycle of pH 5.5 to 7.4. (**B**) Absorption peaks at each pH cycles extracted from (**A**). (**C**) Particle sizes and (**D**) surface potentials of the particles at each cycles of pH. Error bars refer to standard deviations from three replicates.

The reversibility of pH induced assembly, and disassembly also has been confirmed by the absorption spectroscopy (Fig. 4A,B) and size measurements (Fig. 4C). When the solution pH was returned to normal physiological pH (~7.4), the absorption spectrum of AuNPs has returned to its original shape implying that the clusters were disassembled and this behavior was well correspondent with the measured particle sizes. This reversible assembly and disassembly behavior of the CytC/ssDNA-AuNPs and correspondingly repeated surface charge variations (Fig. 4D) remained reproducibly over several repeated experiments with pH changes. These results imply that the synthesized and delivered nanoparticles have a potential of showing repeated therapeutic effects without additional introduction of the nanoparticles into a body.

**Figure 5:**
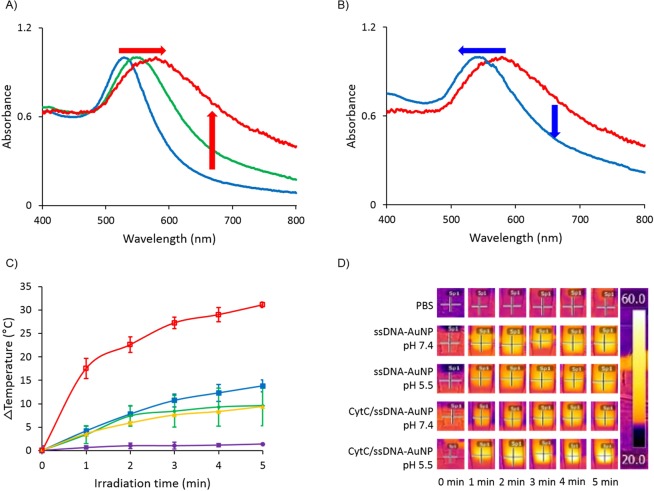
(**A**) Red and (**B**) blue shift of absorption peak accordingly with reducing and elevation pH in DMEM media solution. (**C**) Solution temperature changes with 660 nm laser irradiation times. Purple: PBS blank solution, yellow: ssDNA-AuNP in pH 7.4 DMEM media, green: ssDNA-AuNP in pH 5.5 DMEM media, blue: CytC/ssDNA-AuNP in pH 7.4 DMEM media, and red: CytC/ssDNA-AuNP in pH 5.5 DMEM media. (**D**) IR camera images of laser irradiated nanoparticle solutions. Error bars refer to standard deviations from three replicates.

In an environment with high salt concentration such as cytoplasm or biological fluid, the electrostatic interaction between the CytC/ssDNA-AuNPs can be relatively diminished compared with that in deionized water^30^. In such case, pH responsive behavior of assembly and disassembly of the synthesized particles also may be deactivated due to the charge shielding. To show the feasibility of the CytC/ssDNA-AuNPs in high ionic strength environment, pH responsiveness of the particles has been evaluated in a cell culture media. The particles were suspended in a DMEM media containing serum and penicillin with the same concentration as in deionized water for the experiment. According to the results (Fig. 5A,B), the CytC/ssDNA-AuNPs remain their pH responsive behavior even in a cell media showing plasmonic absorption peak shift accordingly with the pH variations. The absorption peak wavelength of the particles in media was 520 nm at pH 7.8 which meant that the particles existed individually. As the pH of the media was lowered to 5.5, the absorption peak shifted to 550 nm immediately and further red shifted to 600 nm in 24 hours of incubation. When the pH has been raised back to 7.4 by adding NaOH solution to the media, the absorption peak has been shifted to 550 nm implying that the assembled nanoparticles were not completely but partially disassembled. This partial disassembly of nanoparticles may have attributed to the charge screening effect of ions on the ssDNA and cytochrome c of the gold nanoparticles.

The plasmonic absorption peak shift to the longer wavelength induced by CytC/ssDNA-AuNPs’ aggregation in acidic pH has a benefit for photothermal therapy of cancer cells. To demonstrate the higher photothermal effect of CytC/ssDNA-AuNPs over non-pH responsive gold nanoparticles, firstly, temperature increase of cell culture media in presence of nanoparticles has been monitored. The media temperatures were measured using a thermal imaging camera for 5 minutes of laser (660 nm, 4 W/cm^2^) exposure time at two different pHs, 7.4 and 5.5. As shown in temperature profiles and thermal camera images in Fig. 5C,D, the temperature of media containing CytC/ssDNA-AuNPs in a pH condition adjusted to be acidic, 5.5, has been elevated more than 30 °C after 5 minutes of laser illumination on the solution. In contrast, temperature increase was not significant in other media. More specifically, a media containing the same nanoparticles, CytC/ssDNA-AuNPs, in pH 7.4 and medias containing ssDNA-AuNPs in both pH 7.4 and 5.5 did not show significant temperature increase even after 5 minutes of illuminations. In those media, the temperature increase reached only about 9~12 °C. The photothermal conversion efficiency (η) of CytC/ssDNA-AuNPs and ssDNA-AuNPs in both pH 7.4 and 5.5 was evaluated. The η values were calculated by the following Eqs. (1) and (2)^31,32^;

1 $${\rm{\eta }}=(h{\rm{S}}({T}_{\max }-{T}_{\max })-{Q}_{dis})/I(1-{10}^{-{\rm{A660}}})$$

2 $$h{\rm{S}}={m}_{D}{C}_{D}/\tau {\rm{S}}$$

where ℎ is heat transfer coefficient, S is the surface area of the container, *T*_*max*_ is the maximum steady temperature of the nanoparticle solution, *T*_*sur*_ is the environmental temperature, is the heat dissipation from the light absorbed by the solvent and container, I is the laser power, A660 is the absorbance of the particles at 660 nm, m_D_ and C_D_ are the mass and heat capacity of the solvent, and τ is the sample-system time constant. According to the calculation, the conversion efficiencies of CytC/ssDNA-AuNPs are 17.8% and 41.2% at pH 7.4 and 5.5, respectively. The efficiencies of ssDNA-AuNPs are 21.7% and 18.5% at pH 7.4 and 5.5, respectively.

**Figure 6:**
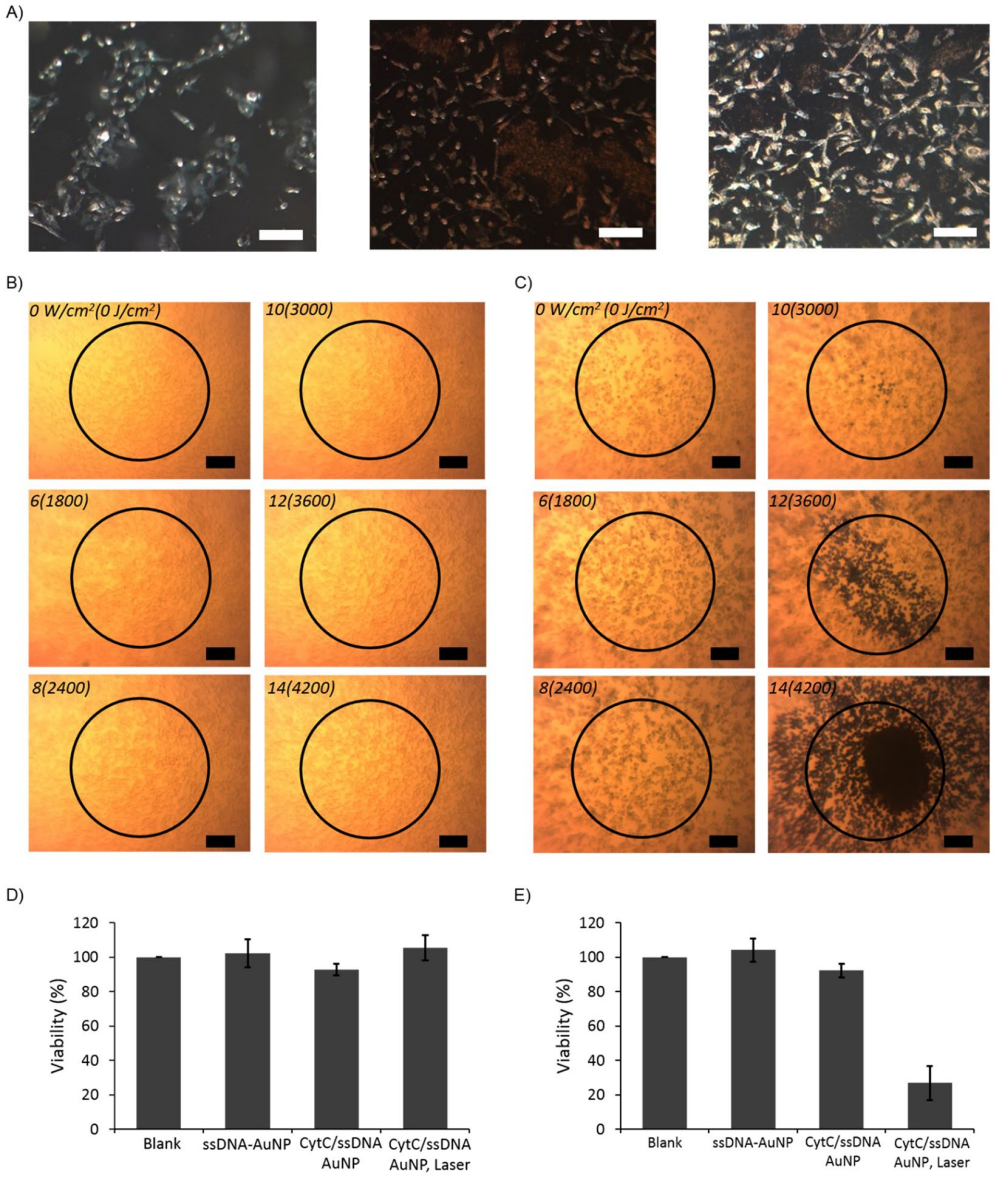
(**A**) Dark field microscope images of B16F10 cells (left), co-incubated with ssDNA-AuNP (middle), and CytC/ssDNA-AuNP (right). Photothermal destruction of the cells co-incubated with (**B**) ssDNA-AuNP and (**C**) CytC/ssDNA-AuNP for 12 h followed by laser irradiation for 5 min at different power densities (scale bar: 100 µm). Viabilities of (**D**) MDCK-GFP cells and (**E**) B16F10 cells co-incubated with each particle with no laser or with laser irradiation.

The high photothermal efficiency of CytC/ssDNA-AuNP in acidic condition can be directly reflected to a photothermal therapy of cancer cells of which pH is lower than that of normal physiological value. Here, the nanoparticles were applied to B16F10 skin melanoma cells and ssDNA-AuNPs were used as a control. The *in vitro* phototherapeutic effect has been evaluated for both of the particles by monitoring the dead cells after illuminating laser light on each of the cells adhered on a culture plate. Before the photothermal therapy, the cellular uptake abilities of the particles were evaluated using dark field microscopy. As seen in Fig. 6A, the pH sensitive CytC/ssDNA-AuNPs were seemed to be accumulated within the cancer cells with remarkably higher efficiency compared with ssDNA-AuNPs. These pH sensitive clustering of CytC/ssDNA-AuNPs and corresponding high accumulation within the cell can contribute to photothermal therapeutic efficiency. The laser has been illuminated on the cells incubated in a presence of 20 nM of nanoparticles and the power has been increased from zero to 14 W/cm^2^ stepwise. As shown in Fig. 6B, the B16F10 cells incubated with ssDNA-AuNPs have not shown any dead cells which are supposed be blue colored by staining with Trypan blue at any of the laser power illuminated on the cells. In contrast, the cells co-incubated with CytC/ssDNA-AuNPs for 24 hours were killed by laser illumination with power of higher than 10 W/cm^2^ (Fig. 6C). At 12 W/cm^2^ of the laser power, almost of the cells positioned within the illuminated area were killed by the photothermal effect. Furthermore, the cells even out of the illumination range were observed to be dead with 14 W/cm^2^ of laser power which is might be attributed to media overheated with high photothermal effect of CytC/ssDNA-AuNPs. In addition, cytotoxicity tests on normal (MDCK-GFP) and cancer (B16F10 melanoma) cells were performed separately to further confirm that the cancer cell selective death was entirely due to the photothermal effect of pH sensitive CytC/ssDNA-AuNPs. Each cell was treated with blank buffer, ssDNA-AuNP, CytC/ssDNA-AuNP, and CytC/ssDNA-AuNP with laser irradiation (14 W/cm^2^ for 5 min), respectively. As shown in Fig. 6D,E, normal cells did not response to any of the stimuli and showed 100% cell viability for all cases. The cancer cells, however, were killed only by the combined treatment of CytC/ssDNA-AuNP with laser irradiation. These results clearly demonstrated the superior photothermal efficiency and therapeutic applicability of the CytC/ssDNA-AuNPs as cancer cells specific photothermal therapeutic agents.

## Conclusion

In summary, we synthesized pH responsive gold nanoparticles with tunable aggregated pH values and investigated their photothermal therapeutic effect. Since the CytC/ssDNA-AuNPs response to the acidic environment and form aggregated particle clusters, the particles result low pH specific high photothermal efficiency on near infrared radiations. This low pH specific aggregation of the synthesized particles can be potentially employed for cancer targeted therapy. The mechanism of pH responsive behavior based on electrostatic interaction between the particles made it possible to reversibly aggregated or disassembled in accordance with solution pH values. In addition, the aggregation pH values of the particles were able to be controlled by varying the relative amount of ssDNA and cytochrome c on AuNPs. *In vitro* evaluation of photothermal therapeutic effect of the synthesized particles showed that the CytC/ssDNA-AuNPs have superior photothermal therapeutic effect than non-pH responsive particle. We believe that this work provides a simple and general strategy for efficient and safe agents for photothermal therapy.

## Method

### Stepwise synthesis of cytochrome c-single stranded DNA capped gold nanoparticles (CytC/ssDNA-AuNP)

Gold nanoparticles of 10 nm size were synthesized using Turkevich Method^33^. Briefly, 6.25 mL of 20 mM gold(III) chloride hydrate (HAuCl_4_) solution was add to boiling 250 mL deionized water(DI). Subsequently 3.75 ml of 100 mM sodium citrate solution was added and vigorously stirred. The color of the solution changed from yellow to black purple in 5 minutes and finally to red. After that, stirring was continued for two hours while maintaining the temperature, and the temperature was cooled down to room temperature for 30 minutes. After cooling, for concentration of gold nanoparticles solution, 100 K MWCO Amicon filter was used to centrifuge at 5500 RCF for 1 h (AuNP). ssDNA-SH 1 mM and AuNP 100 nM were mixed and stirred for 12 hours. The molar ratio of ssDNA-SH to AuNP was 400: 1. Then 1 M NaCl and 1XPBS were added to the solution to proceed the salt aging process. The amount of 1 M NaCl and 1XPBS are 12.5% of AuNP and ssDNA-SH volume. The 1 M NaCl and 1XPBS were divided into10 groups and put into every 10 times. After that, the solution stirred again for 12 hours and the solution was centrifuged twice at 5500 RCF for 45 min for remove extra ssDNA-SH (ssDNA-AuNP). ssDNA-AuNP 100 nM and cytochrome c 67 μM solution were mixed and the pH was adjusted to 10.5 using 1 M NaOH. The molar ratio of cytochrome c to ssDNA-AuNP was 1000: 1. Mixture of cytochrome c and ssDNA-AuNP were centrifuged at 5500 RCF for 40 min twice and supernatant was removed. The final CytC/ssDNA-AuNP solution was adjusted to pH 10.5 by using 100 mM NaOH.

### Characterization CytC/ssDNA-AuNP

Ultraviolet visible (UV/Vis) spectroscopy was used to measure the absorption spectrum of citrate-, ssDNA functionalized, ssDNA and cytochrome c functionalized gold nanoparticles at diverse pH values. For UV/Vis spectroscopy 0.2 ml of a diluted solution of gold nanoparticles at each synthesis step was transferred into a disposable cuvette, and absorption spectrums were obtained using a Biospectrometer (Eppendorf, Germany). The stability of CytC/ssDNA-AuNP was measured in the solutions of deionized (DI) water, 100 mM NaCl, 1XPBS, Serum involved DMEM. 20 nM of CytC/ssDNA-AuNP in each solution was incubated at different time (0, 2, 6, 12 hours) and absorption spectrum was obtained using a Biospectrometer (Eppendorf, Germany). DLS was used to estimate the size distribution and zeta potential of citrated, DNA functionalized, DNA and Cytochrome C functionalized gold nanoparticles at diverse pH values. For DLS measurements, 0.8 ml of a diluted solution of each construction step gold nanoparticles at several diverse values was transferred into a disposable cuvette, and hydrodynamic size and zeta potential measurements were obtained using a Zetasizer Nano (Malvern, UK). Gel electrophoresis was used to confirm that grafted SH-X01, Cytochrome C protein onto Gold nanoparticles. Suspension of AuNP, ssDNA-AuNP, CytC/ssDNA-AuNP in the presence of 10% glycerol were loaded on 0.5% agarose gel in 0.5X Tris-borate-ethylendiamineteraacetic acid (EDTA) buffer (TBE buffer) and run in a Wide Mini-Sub Cell GT Horizontal Electrophoresis System (Biorad) at 100 V for 60 min. For measure SH-X01 number on surface of AuNP, QuantiFluor ssDNA(Promega, USA) was used. The dye stained extra SH-X01 in the supernatant of CytC/ssDNA-AuNP and fluorescence spectrometer (FS-2, Sinco) was used to measure fluorescence of stained dye. The excitation wavelength is 492 nm and observed fluorescence at 528 nm. And compared with initial SH-X01 that was used to assemble ssDNA-AuNP. To measure cytochrome c numbers on surface of AuNP we obtained absorption spectrum from Biospectrometer. Comparing the CytC/ssDNA-AuNP supernatant with the absorbance spectrum of the initially used Cytochorme C that was used to assemble CytC/ssDNA-AuNP at 410 nm, we obtained the Cytochrome C concentration.

### Temperature monitoring of CytC/ssDNA-AuNP

To confirm the temperature increasing effect of CytC/ssDNA-AuNP by laser, the 660 nm red diode laser module was used to irradiate on 20 nM of Cyt/ssDNA-AuNP, ssDNA-AuNP, 1XPBS at SERUM involved DMEM by changing pH values (7.4, 5.5). 660 nm red diode laser module 1 W (LSR660NL-1W, Deal Mar Photonics) was irradiated for 5 minutes on 20 mM AuNP, ssDNA-AuNP, CytC/ssDNA-AuNP 400uL. Thermal imaging camera C2(FLIR) was used to measure Temperature changing.

### SEM imaging

Field emission scanning electron microscope (FE-SEM) was used to confirm the morphology of particles. AuNP, ssDNA-AuNP, CytC/ssDNA-AuNP was loaded 1 nM 10uL on Carbon template and they are dried in petri dish for 6 hours at room temperature. They are observed by SU-70, (Hitachi) at 15.0 kV.

### TEM imaging

AuNP, ssDNA-AuNP, CytC/ssDNA-AuNP was loaded 1 nM 12uL on carbon coating copper grid (Ted Pella) and they were dried for 24 hours at room temperature. They were observed by JEM-2100, (JEOL) at 200 kV.

### Cell experiments

B16 F10 mouse melanoma cells were purchased from Korean Cell Line Bank. B16 F10 cells were incubated in Dulbecco’s Modified Eagle Medium (DMEM, HyClone) with 10% FBS and 1% PS. Cells were grown onto 12 mm glass coverslips (for dark field imaging or confocal microscope imaging) or directly (for *in vitro* photo thermal therapy) in a 24 well plates at a density of 1 × 10^5^ cells/well at 37 °C under 5% CO_2_. After 1 days, cells were incubated with CytC/ssDNA-AuNP, ssDNA-AuNP were used as control groups. Cells were rinsed with culture media and exposed to laser illumination for *in vitro* photo thermal therapy. Then they were stained with 0.4% trypan blue for 5 min to test cell viability.

### Cytotoxicity test

B16 F10 melanoma cell and MDCK-GFP cell suspension (10000 cells/well) was dispensed in a 96-well plate (Corning) and incubated for 12 h at 37 °C under 5% CO_2_. Then, cells were co-incubated with 20 nM CytC/ssAuNPs-AuNPs and ssDNA-AuNPs for 12 h at 37 °C under 5% CO_2_. After that, the Cell Counting Kit-8 solution (CCK-8, Dojindo Laboratories, Kumamoto, Japan) was added to each well of the plate. After further incubation for 2 h, absorbance at 450 nm was measured using a microplate reader. Results are expressed as the ratio of the absorbance of the positive control, no gold nanoparticle treated cells. To determine the cancer ablation rate with CytC/ssDNA-AuNPs under light irradiation, B16 F10 melanoma cell suspension (10000 cells/well) was dispensed in a 96-well plate (Corning) and incubated for 12 h at 37 °C under 5% CO_2_. Then, cells were co-incubated with 20 nM CytC/ssAuNPs-AuNPs for 12 h at 37 °C under 5% CO_2_. After that, 660 nm laser with 14 W/cm^2^ was irradiated to each well of the plate for 5 min and the Cell Counting Kit-8 solution (CCK-8, Dojindo Laboratories, Kumamoto, Japan) was added to each well of the plate. After further incubation for 2 h, absorbance at 450 nm was measured using a microplate reader. Results are expressed as the ratio of the absorbance of the positive control, no gold nanoparticle treated cells.

## Supplementary information

Supplementary information.
