# Supplementary material for: Reversibly pH-responsive gold nanoparticles and their applications for photothermal cancer therapy
Source: Sci Rep. 2019 Dec 27;9:20180. doi: 10.1038/s41598-019-56754-8 (PMC6934723; doi:10.1038/s41598-019-56754-8)
Supplement: Supplementary file 1 — Supplementary information. [file 41598_2019_56754_MOESM1_ESM.pdf]

Supplementary Information

Reversibly pH-responsive gold nanoparticles and their applications for photothermal cancer therapy

Sanghak Park[1,†], Woo Jin Lee[2,†], Sungmin Park[1], Doowon Choi[2], Sungjee Kim[2,*], and Nokyoung Park[1,*]
[1]Department of Chemistry, Myongji University, 116 Myongji Ro, Yongin, Gyeonggi-do, South Korea.
[2]Department of Chemistry, POSTECH, 77 Cheongam Ro, Nam Gu, Pohang, South Korea.

Materials section

Materials

Gold(III) chloride hydrate, sodium citrate dihydrate, cytochrome c from bovine heart, hydrochloric acid, sodium hydroxide, phosphate buffered saline tablet, agarose was purchased from Sigma(USA). 36 bases thiol modified single stranded DNA (ssDNA-SH, Table S1) was commercially synthesized by Integrated DNA Technologies (Skokie, IL, USA). TE (pH 8.0), 50XTAE, 5XTBE Buffer, 5 M NaCl solutions were purchased from Bioneer (South Korea).

Supplementary Data

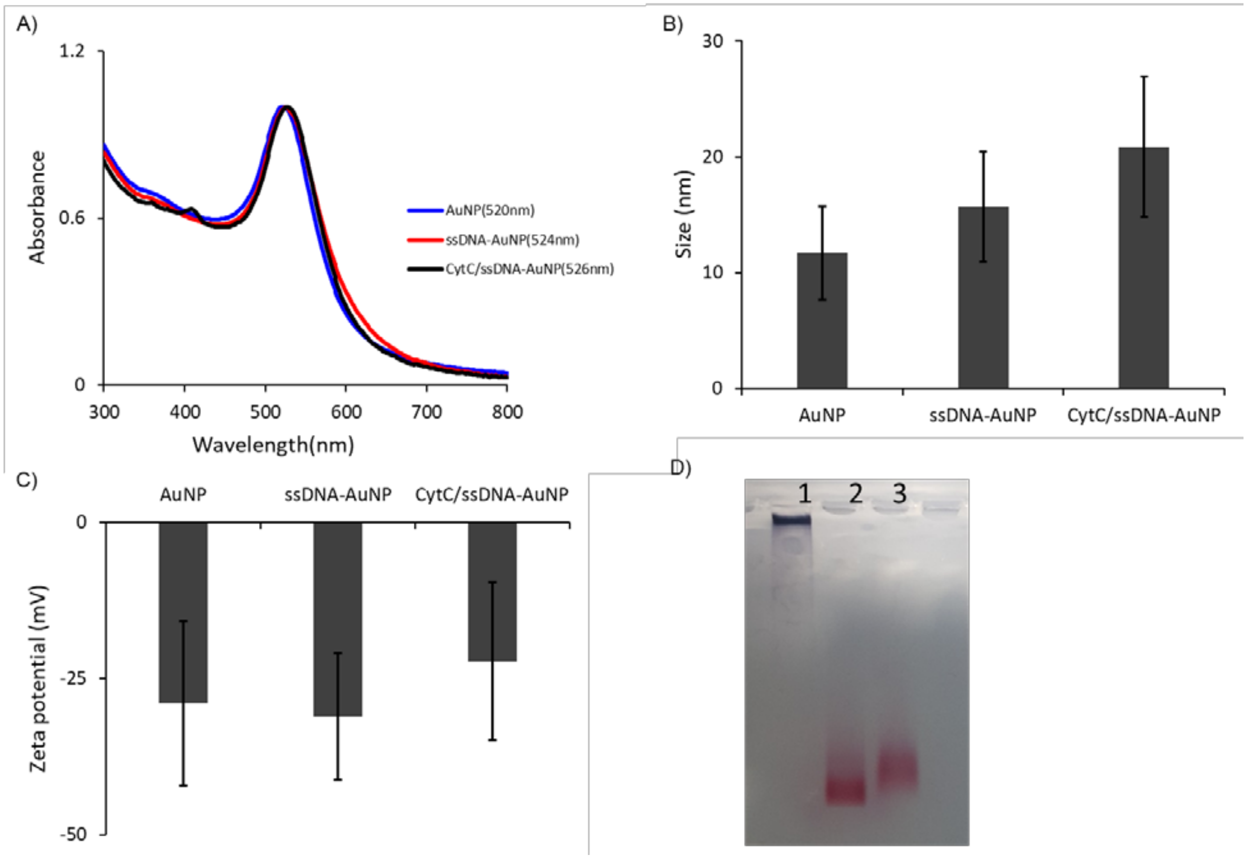

Figure S1. Characterization of the stepwise synthesis of CytC/ssDNA-AuNP. (A) Absorption spectra, (B) Size distributions, (C) Surface charges and (D) Gel electrophoresis mobility of AuNP (Lane 1), ssDNA-AuNP (Lane 2), and CytC/ssDNA-AuNP (Lane 3), respectively.

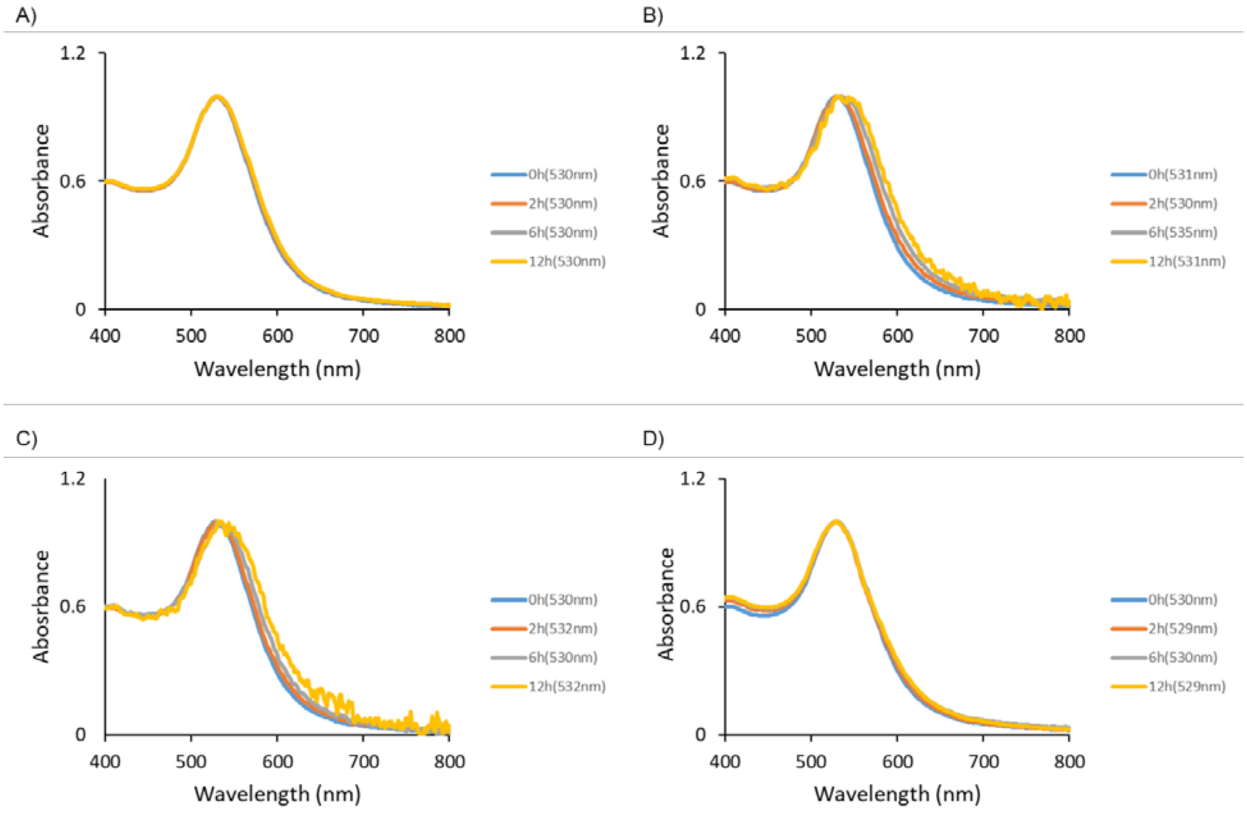

Figure S2. Stability evaluation of CytC/ssDNA-AuNP in (A) DI water, (B) 100 mM NaCl, (C) 1xPBS, and (D) DMEM media including serum.

Table S1. Physical properties of CytC/ssDNA-AuNP with lowering the pH.

	pH 7.4	pH 6.5	pH 6	pH 5.5
Maximum absorption peak (nm)	526	548	562	575
Hydrodynamic size (nm)	24.7(±0.3)	64.7(±1.2)	411.4(±11.5)	592.6(±23.8)
Zeta potential (mV)	-25.8(±7.6)	-18.1(±7.1)	-5(±5.5)	+2.6(±4.8)

Table S2. Physical properties of CytC/ssDNA-AuNP with raising the pH.

	pH 5.5	pH 6	pH 6.5	pH 7.4
Maximum absorption peak (nm)	575	562	548	528
Hydrodynamic size (nm)	592.6(±23.8)	65(±18.5)	51.7(±9.2)	36.2(±12)
Zeta potential (mV)	+2.6(±4.8)	-5.2(±8.2)	-17.2(±7.6)	-19.3(±7.3)

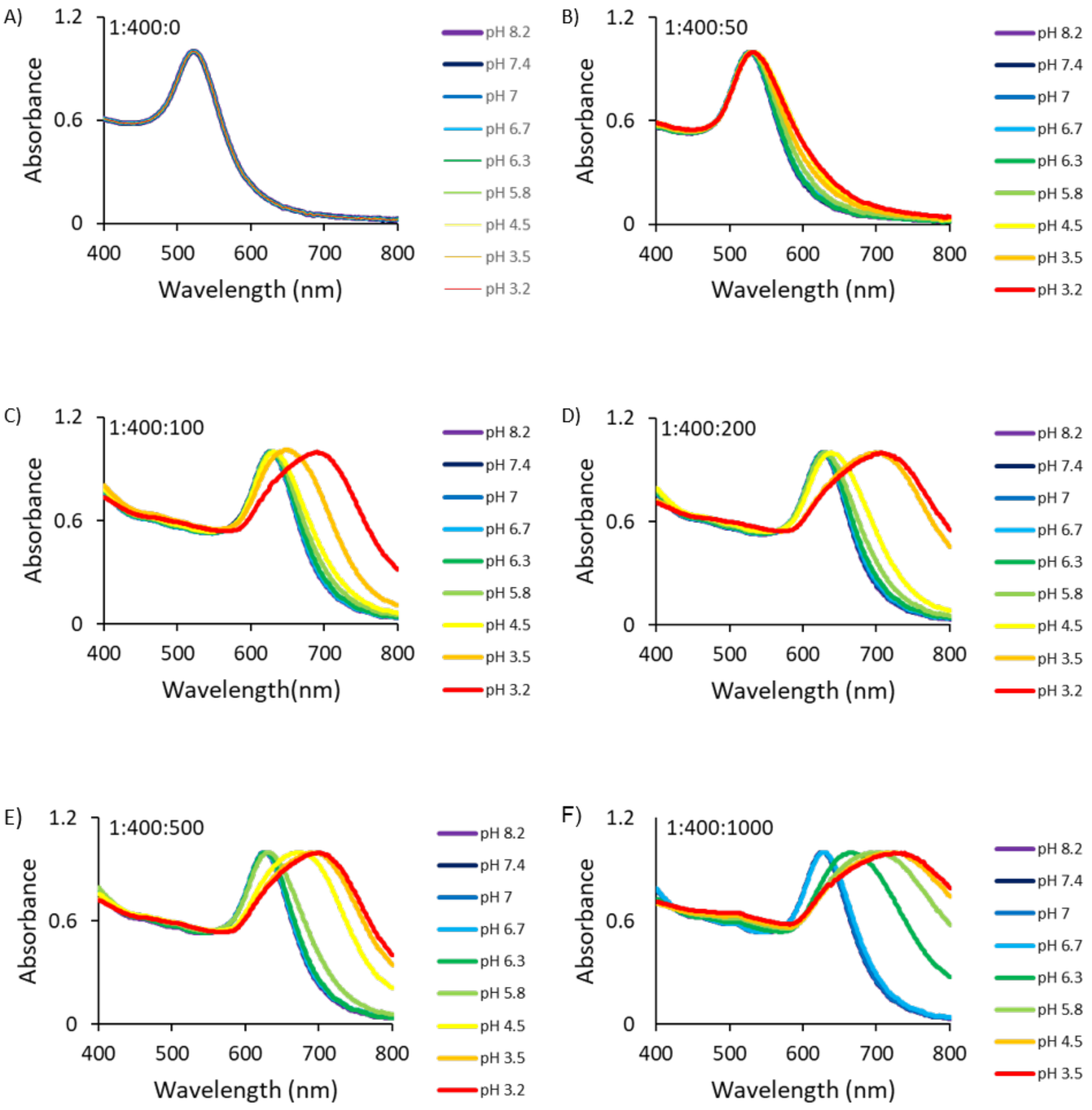

Figure S3. Absorption spectrum peak shifts of CytC/ssDNA-AuNPs in various pHs accordingly with the reaction ratios of AuNP vs. ssDNA vs. cytochrome c as indicated in each graph.

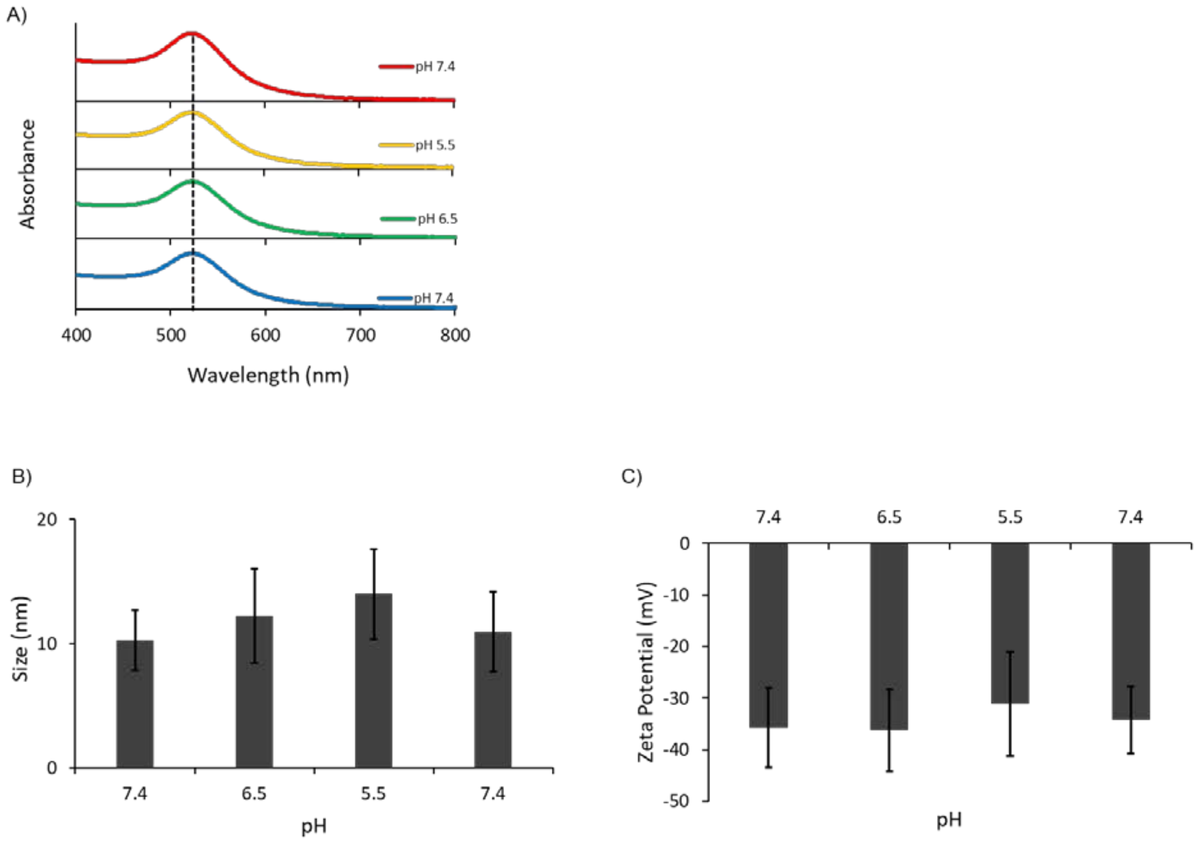

Figure S4. (A) Absorption spectra, (B) Size distribution, and (C) Surface charges of ssDNA-AuNP in various pHs; 7.4→6.5→5.5→7.4.

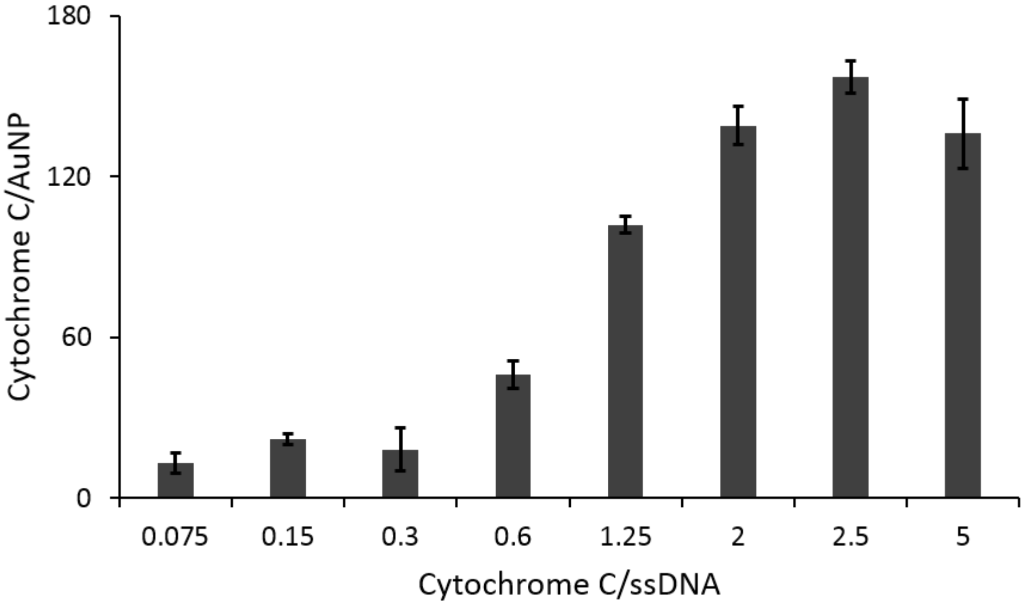

Figure S5. The number of cytochrome c on a single AuNP associated with reaction ratio of cytochrome c and ssDNA.

Table S3. The number of cytochrome c and ssDNA on a single AuNP associated with reaction ratio of cytochrome c and ssDNA

Reaction ratio (Cytochrome C/ssDNA)	Cytochrome C/AuNP	ssDNA/AuNP
0.075	13(±4)	N/A
0.15	22(±2)	N/A
0.3	18(±8)	N/A
0.6	46(±5)	N/A
1.25	102(±3)	N/A
2.0	139(±7)	338(±4)
2.5	157(±6)	294(±19)
5	136(±13)	159(±8)
